# p53 biology and reactivation for improved therapy in MDS and AML

**DOI:** 10.1186/s40364-024-00579-9

**Published:** 2024-03-13

**Authors:** Joanna E. Zawacka

**Affiliations:** 1 Department of Oncology-Pathology, Karolinska Institute, Stockholm, Sweden; 2 Department of Biochemistry, Laboratory of Biophysics and p53 Protein Biology, Medical University of Warsaw, Warsaw, Poland

**Keywords:** MDS, AML, p53, MDM2, MDM4, p73, Improved therapy

## Abstract

Myelodysplastic syndrome (MDS) and acute myeloid leukemia (AML) originate from preleukemic hematopoietic conditions, such as clonal hematopoiesis of indeterminate potential (CHIP) or clonal cytopenia of undetermined significance (CCUS) and have variable outcomes despite the successful implementation of targeted therapies. The prognosis differs depending on the molecular subgroup. In patients with *TP53* mutations, the most inferior outcomes across independent studies were observed. Myeloid malignancies with *TP53* mutations have complex cytogenetics and extensive structural variants. These factors contribute to worse responses to induction therapy, demethylating agents, or venetoclax-based treatments. Survival of patients with biallelic *TP53* gene mutations is often less than one year but this depends on the type of treatment applied. It is still controversial whether the allelic state of mutant *TP53* impacts the outcomes in patients with AML and high-risk MDS. Further studies are needed to justify estimating *TP53* LOH status for better risk assessment. Yet, *TP53*-mutated MDS, MDS/AML and AML are now classified separately in the International Consensus Classification (ICC). In the clinical setting, the wild-type p53 protein is reactivated pharmacologically by targeting p53/MDM2/MDM4 interactions and mutant p53 reactivation is achieved by refolding the DNA binding domain to wild-type-like conformation or via targeted degradation of the mutated protein. This review discusses our current understanding of p53 biology in MDS and AML and the promises and failures of wild-type and mutant p53 reactivation in the clinical trial setting.

## Introduction

**Fig. 1:**
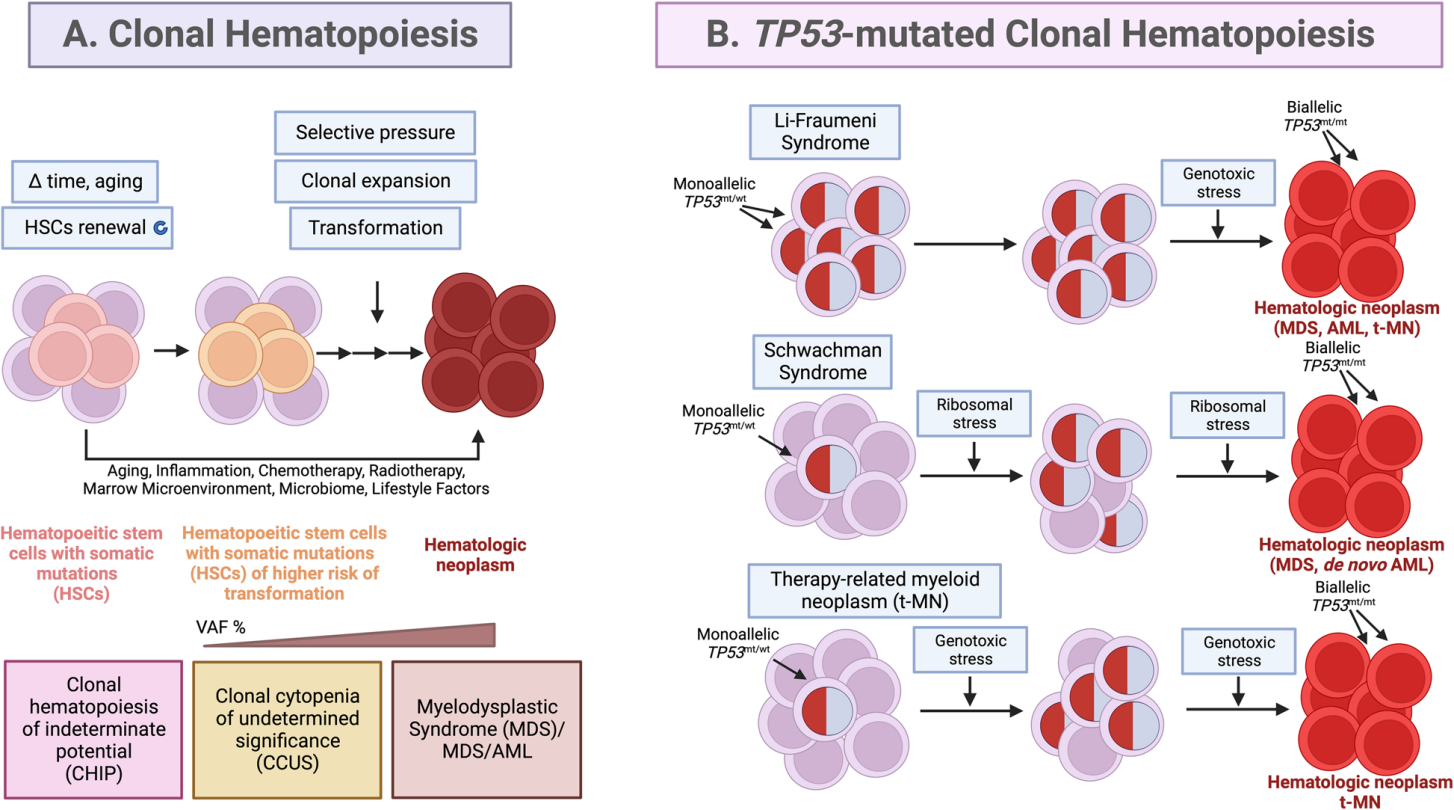
Origin of hematological neoplasms from clonal hematopoiesis (**a**) and *TP53*-mutated clonal hematopoiesis (**b**). With age, hematopoietic stem cells (HSCs) acquire somatic mutations and the mutated clones are expanded during HSCs renewal. Early genetic events lead to clonal hematopoiesis (CH) and the origin of clonal hematopoiesis of indeterminate potential (CHIP) or clonal cytopenias of undetermined significance (CCUS), which have different genetic backgrounds, differ in cytopenia status but possess same variant allele frequencies of VAF ≥ 2%. Among these, CCUS have a higher potential of transformation to myelodysplastic syndrome (MDS) with a range of incidence 18–95%. Fitness variants occurring later, confer a growth advantage in the presence of selective pressures such as inflammation, cytotoxic treatment, radiotherapy, bone marrow microenvironment or aging. The consequent selection of high-risk variants, accumulation of the co-occurring mutations, increase in the VAF > 10% and the co-existing cytopenia are predictive of hematopoietic malignancy development and thus, accelerate the progression to hematological neoplasm; myelodysplastic syndrome (MDS) and MDS/AML. *TP53*-mutated CH occurs in about 2–6% of cancer patients. *TP53*-mutated MDS and AML account for up to 13% of all de novo cases. In therapy-related myeloid neoplasm, *TP53* gene mutations are present in 20–40% of cases [5]. The model shows the pathogenesis of MDS and AML in hereditary cancer syndromes; Li-Fraumeni syndrome (LFS) with congenital *TP53* mutations and Schwachman syndrome (SDS) with congenital mutations in Schwachman–Bodian–Diamond syndrome (*SBDS*) gene and in therapy-related myeloid neoplasm (t-MN); diseases in which *TP53* monoallelic mutations play a role in the progression from CH to myeloid malignancy. The incidence of malignant transformation is higher in the presence of selective pressure as delineated for SDS and t-MN. →  →  → tandem of arrows indicates a multi-step process. Modified from [1, 6, 7]. Created with BioRender.com

Myelodysplastic syndromes (MDS) and acute myeloid leukemia (AML) are cognate, clonal hematological neoplasms and originate from pre-malignant, mutated hematopoietic stem cells (HSCs) that undergo clonal expansion after selection pressure, in a process called clonal hematopoiesis (CH) [1]. Extensive studies showed HSCs are constricted to the lineage − CD34 + CD38 − CD90 + CD45RA − compartment and bear driver mutations in CH [2]. CH results in an accumulation of large numbers of abnormal, immature myeloid cells in the bone marrow and peripheral blood called leukemic stem cells (Fig. 1a). Clonal hematopoiesis often occurs due to aging and is associated with a higher risk of hematological cancers. The rate of CH progression to hematologic neoplasm is 0.5%—1% per year [3, 4].

During their lifespan, hematopoietic stem cells (HSCs) experience functional decline due to accumulated mutations resulting from increased DNA damage or epigenetic reprogramming. They reside in the bone marrow as a genetically heterogeneous cell population [8]. Some HSCs that acquire somatic mutations in genetic modulators (*DNMT3A*, *TET2*, *ASXL1*) and in signaling molecules (*JAK2V617F*) gain a competitive fitness advantage in the presence of selective pressure and expand resulting in clonal hematopoiesis [9, 10].

MDS and AML originate during CH from clonal hematopoiesis of indeterminate potential (CHIP) or clonal cytopenia of undetermined significance (CCUS), which falls into the category of clonal cytopenia (Fig. 1a). CCUS can be distinguished from CHIP thanks to the advancements in NGS techniques. It represents a continuum with MDS to which it progresses faster than CHIP after acquiring additional mutations and dysplasia [6, 11]. In both, CHIP and CCUS the increased risk of progression to MDS and de novo AML occurs upon > 1 additional driver mutation, VAF > 10% and acquisition of additional mutations [12]. For patients with CCUS, the risk of progression to MDS/AML was reported to be 18% within 16 months and 95% in 10 years [3].

Many MDS and AML subtypes share common driver alterations which might occur at different frequencies but target the same pathways; for example DNA methylation (*TET2*, *DNMT3A*, and *IDH1/IDH2*), chromatin/histone modification (*MLL2*, *EZH2* and *ASXL1*), RNA splicing (*SF3B1*, *SRSF2*, *U2AF1*, *U2AF2*, and *SF3A1*) or have mutations in *TP53* gene, in *RAS* gene or in other signaling pathways [13, 14].

Somatic mutations in *TP53* are considered early events in leukemogenesis and blood stem cells with *TP53* mutations constitute a preleukemic niche. The pre-leukemic stem cells (pre-LSCs) with *TP53* mutations retain the ability to differentiate which highlights the role of *TP53* mutations in leukemogenesis [15] as described in more detail below.

Studies showed that in patients with therapy-related AML or secondary AML, the analysis of the material from antecedent hematological disorder displayed mutations in *TP53* which manifests the role of low levels of the *TP53* mutated clone in the disease origin, before the cytotoxic treatments for the primary malignancy [16]. The material from precedent disorder and/or CH or CCUS is not always available for MDS and AML patients, and early genetic alterations, like somatic *TP53* mutations, cannot be tracked back, limiting the feasibility of applying mutant p53 as a prognostic biomarker in therapy-related AML or secondary AML.

MDS and AML are yet, heterogenous bone marrow disorders with common characteristics like expansion of clonal hematopoietic stem cells, cytopenia and marrow dysplasia.

### Myelodysplastic syndrome (MDS)

MDS, especially high-grade MDS, has a high risk of transformation to a secondary AML [17]. MDS is the most common adult myeloid malignancy, has a blast count < 20%, and in about 30% transforms to AML, termed “secondary AML to MDS” or bone marrow failure. According to the International Consensus Classification (ICC), the novel intermediate state, MDS/AML, is characterized by the presence of ≥ 10% but < 20% of blasts which is applied to show a continuum of MDS to AML [9, 18]. The new, 2022, classification systems, ICC and WHO 2022, of MDS and AML, though accurate in genetic markers classification and in regression of blast threshold, is troublesome for healthcare providers due to existing discrepancies in subclassification and diagnostic criteria of acute myeloid leukemias which might affect the choice of standardized treatment or clinical trial eligibility [18]. Yet, it is outside the scope of this review to discuss the clinical weight and the shortcomings of the current classification system. Generally, MDS patients have a poor prognosis, with a median overall survival of only 5 years. MDS patients who progressed to AML, have higher-risk MDS, with myeloblast count ≥ 20% and acquired/expanded abnormalities in *TP53*, *RUNX1*, or *RAS* genes [19]. Phenotypically, high-grade MDS has lower cell death rates when compared to lower-risk patients [20], and typically has inferior rates of complete remission, relapse-free survival, and overall survival compared with patients with de novo AML [19].

### Acute myeloid leukemia (AML)

Chromosomal abnormalities, copy number variations and translocations and inversions are common genetic events in both, MDS and AML. AML is on average diagnosed in older patients, 68 years old or older, and has poor outcomes with the five-year overall survival of less than 30% and up to 50% in younger patients [21]. Regardless of the age of diagnosis, patients who have not responded to induction therapy, have dismaying outcomes [6]. The criterion for AML diagnosis might differ depending on the driver mutation, yet in the majority, AML patients have ≥ 10% or 20%, if misdiagnosis with chronic myeloid leukemia (CML) might occur. Secondary AML accounts for up to 25% to 35% of total AML cases [19] with most (60–80%) arising from MDS. Due to clinical impact, in the new ICC system, a new subgroup was generated which constitutes a separate entity within the group of myeloid neoplasms with mutated *TP53* which includes MDS, MDS/AML and AML with mutated *TP53* [9, 22, 23]. *TP53* mutations underly the aggressiveness of AML and, even though in MDS multihit *TP53* mutations are required for diagnosis of MDS with mutated *TP53*, in AML and MDS/AML with mutated *TP53*, any pathogenic *TP53* mutation VAF of ≥ 10% is sufficient for diagnosis [24]. *TP53* mutations, both clonal and subclonal (VAF < 20%), have been firmly associated with an adverse outcome, as supported by significantly inferior complete remission, overall survival, and event-free survival rates [25]**.**

Patients with AML having subclonal *TP53* mutations are less likely to have complex karyotype or chromosomal losses. However, the responses to treatment were poor regardless of the mutation type. It was observed that the *TP53*-mutated cohort had a significantly lower complete remission rate (below 50%) compared to *TP53* wild-type patients (above 80%). This rate was equally distributed among the VAF-based groups (*TP53* VAF > 40%, 20%-40%, and < 20%). The 3-year overall survival rate had notable differences between *TP53* wild-type and *TP53*-mutated patients (49.1% vs 8.3%) [25].

In 2017 and later, eleven, new drugs or combinations were approved for AML by the Food and Drug Administration [26]. Among the new approvals, five drugs target known AML vulnerabilities; FLT3 (midostaurin [27], gilteritinib [28]), IDH1 (ivosidenib) [29], IDH2 (enasidenib) [30] and BCL2 (venetoclax) [31]. Yet, the targeted treatments are not effective in high-risk *TP53*-mutated AML patients, who have dismaying outcomes as assessed for the frontline treated patient group [32].

### p53 tumor suppressor protein

P53 is a tumor suppressor and is encoded by the *TP53* gene located at 17p13.1, a site undergoing chromosomal aberrations resulting in cytogenetic deletion at 17p13.1; loss of heterozygosity (LOH) at the 17p *TP53* locus or mutations largely of missense type [22]. *TP53* gene (coding for p53 protein) is often mutated in human cancers, in the majority in the DNA binding domain. Cancers with a high incidence of *TP53* mutations are high-grade serous ovarian cancer, lung, colon, brain or pancreatic cancer [33]. The *TP53* gene mutations of missense, nonsense, frameshift or in/dels types, may result in loss-of-function, gain-of-function or in the dominant negative effect [34] propensities of the mutated protein (reviewed in [35]). Yet, our understanding of the biology behind the multiple pathogenic variants is limited.

In cancers with intact *TP53* gene, the functional protein is inactivated by overexpressed mouse double minute 2 (MDM2) and/or MDM4 which bind to the N-terminal domain and inhibit the transcriptional function of p53, or promote p53 mono- and/or polyubiquitination and nuclear export and proteasomal degradation [33]. p53 is a transcription factor which directly or indirectly activates or represses a range of target genes involved in a multitude of cellular processes like; cell cycle regulation, DNA repair, senescence, pro- and anti-oxidant response, apoptosis, ferroptosis, pyroptosis, cuproptosis, autophagy, immune response, inflammation, metabolism or fertility and stem cells' renewal. The decision by which p53 drives the cell response to stress stimuli is complex and depends on the post-translational modifications and tissue/cell context (reviewed in [36]). The non-transcriptional functions of p53 are currently under debate and will not be discussed in this review.

In MDS and AML, p53 protein inactivation and *TP53* gene mutations represent a clinically important predictive biomarker to DNA damaging chemotherapy [37] or venetoclax [38, 39] and are thus, underlying adverse prognoses which may dependent on complex karyotype.

In a clinical study called VIALE-A, *TP53* mutant and wild-type AML patients who were new to treatment were given venetoclax and azacytidine (AZA) or AZA alone. Results showed improved remission rates but no significant difference in duration of response (DoR) or overall survival (OS) with the combination therapy in patients with poor-risk cytogenetics and mutant *TP53*. *TP53* wild-type and complex karyotype patients showed comparable remission rates, DoR, and OS to the patients with intermediate-risk cytogenetics.

This review will highlight the contemporary status of the p53, p53-activating drugs and emerging new, investigational therapies targeting the p53 in MDS and AML.

### *TP53* gene alterations in MDS and AML

MDS and MDS/AML develop from mutated clones present in the hematopoietic compartment [40]. Yet, the presence of a *TP53*-mutated clone alone is not sufficient for the development of effective leukemogenesis (Fig. 1b).

Within European Leukemia Net (ELN) 2017, *TP53* mutations are associated with unfavorable risk category and decreased overall survival (OS < 2 years). Patients with AML with *TP53* mutations and complex karyotype (CK) have inferior OS of 161 days vs 374 days compared with wild-type *TP53* [41]. Even though, patients with mutant *TP53* AML after complete remission receive allogeneic hematopoietic stem cell transplants, yet are among the group with high relapse rates [42]. Patients with MDS with excess blasts-2 (MDS-EB-2) and *TP53* mutations, share similar characteristics and clinical outcomes with mutant *TP53*, de novo AML patients. According to recent reports, both, *TP53* mutated AML and MDS-EB-2, have practically undistinguishable biology; have blast count 15%-20% (20% cutoff is not considered specific any longer), in the majority (50%—70%), have no co-existing driver mutations or rareness of *NPM1* or *FLT3* alterations [24, 43], and possess high incidence of complex/monosomal karyotypes (80%–90%), which include abnormalities in chromosomes 5, 7, and 17 [24]. In therapy-related myeloid malignancies, *TP53* mutations are not induced by the treatment itself but by existing progenitor clones with mutant *TP53*, that are resistant to DNA-damaging therapy, and expand in clonal hematopoiesis to give raise to *TP53*-mutated MDS/AML (Fig. 1b) [1].

Congenital cancer predisposition syndromes predisposing to myeloid neoplasm are a separate entity according to WHO [44]. Hereditary cancer predisposition syndrome, Li- Fraumeni (LFS), is an autosomal dominant condition connected with a high risk of a broad range of childhood- and adult-onset cancers. Patients with LFS develop multiple tumors during their lifespan; predominantly soft tissue sarcomas, osteosarcomas, pre-menopausal breast cancers, brain tumors, adrenocortical tumors and less frequently, pancreatic, ovarian or gastrointestinal cancers and other [45, 46]. The incidence of leukemias is < 5% [47, 48]. LFS is described by the heterozygous germline mutations in the *TP53* gene [49]. Family history of inherited mutant (pathogenic variant) *TP53* is a key criterion for the consideration of LFS yet, de novo mutations occur in ∼10%–20% of LFS cases [50]. Recent whole-genome sequence analysis combined with clock-like mutational signatures and MutationTimeR algorithm revealed that in LFS patients *TP53* LOH occurs many years before tumor diagnosis, likely already in utero. It has been concluded that the copy number gains of mutant *TP53* occur spontaneously in LFS patient cells and can readily outcompete diploid clones in a small number of generations [51]. In LFS patients all hematopoietic progenitor stem cells (HSPCs) carry *TP53* mutations. Yet, the patients may mainly develop treatment-related myeloid neoplasm (t-MN) later in life (Fig. 1b) and prognosticate a poor prognosis with standard therapies and even allogeneic stem cell transplant [47, 52]. Thus, the risk of the development of t-MN in LFS patients should be taken into consideration when administering radiation treatment or myelosuppressive therapy.

Schwachman syndrome (SDS) with congenital mutations in Schwachman–Bodian–Diamond syndrome (*SBDS*) gene is a condition of high risk of developing myeloid neoplasms (MN) early in life [53]. In SDS, the SBDS protein which promotes the formation of the mature, translationally active 80S ribosome, is mutated, resulting in decreased ribosomal subunit joining and reduced translation efficiency and ribosomal stress (Fig. 1b) [54]. Survival is poor in SDS patients who develop MDS or AML originating from CH [55, 56]. It has been reported that the presence, number, persistence, and allele abundance of somatic *TP53* mutations were not predictive of leukemia risk in SDS patients with CH, yet, the progression of *TP53*-mutated clones was found to be driven by the development of bi-allelic alterations of the *TP53* locus via deletion, copy number (CN)-LOH, or point mutation (Fig. 1b) [56]. It is hypothesized that continued ribosome stress in SDS HSPCs carrying a heterozygous *TP53* mutation selects for clones that inactivate the second *TP53* allele and lead to the development of *TP53*-mutated CH, but also to its progression to myeloid malignancy (Fig. 1b) [1].

Because of the adverse outcomes for patients with *TP53*-altered AML/MDS as *per norm*, it should be strongly encouraged to enrol the patients into clinical trials. Such a strategy would let patients access promising treatments or new combinations with the potential to improve outcomes since the current gloom scenario shows dismal median survival only up to 10 months, irrespective of therapies used [57].

### Targeting mutant p53 for improved therapy in MDS and AML

In *TP53*-mutated MDS, the “multihit” involvement with other genomic or chromosomal alterations is observed [58]. *TP53* copy-number loss is prevalent in 70% of AML cases with a concomitant *TP53* gene abnormality [32]. The recently investigated cohort of five hundred de novo and refractory AML patients shows that around 80% of patients harbor missense substitutions in the *TP53* gene. Nonsense or in/del mutations are less common. In frontline patients, the predominant missense variations were R248, R273, R175 and Y220. CN loss with concomitant hot-spot *TP53* variants is more deleterious in comparison with those with normal CN. In the cited work, the authors conducted an integrative multidimensional evaluation. The analysis was based on the interplay of somatic mutations, CN alterations, and protein expression patterns assessed using digital image-assisted immunohistochemistry. The study showed that *TP53* mutation and CN status correlate with p53 protein expression patterns, such as p53^high^ and p53^truncated^, which predict mutant *TP53* [32]. Also, it was demonstrated that the contribution to MDS pathogenesis and AML transformation is more likely to involve *TP53* hotspots that differ from those involved in de novo AML pathogenesis [32].

The most common hot-spot mutations in *TP53* can be classified into two groups; structural mutants which have altered conformation of the DNA binding domain and DNA-contact mutants; which have alterations in amino acids responsible for direct interactions with DNA. Contact mutants like p53-R273H or p53-R248Q display aberrant interactions with DNA as reported by several labs [59, 60], yet, unlike conformational mutants (e.g. p53-R175H), have a structure similar to wild-type protein [61].

**Fig. 2:**
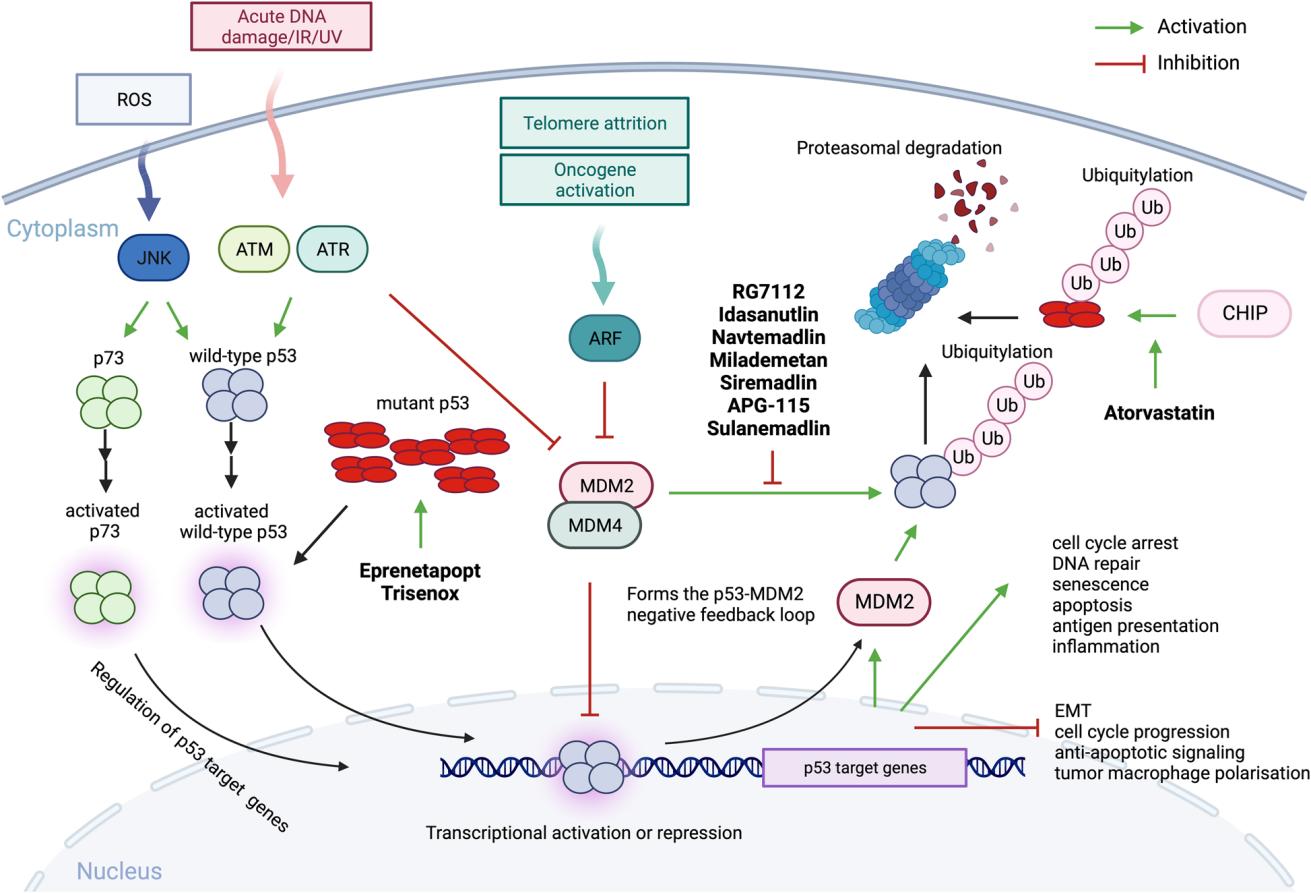
A simplified scheme of p53 regulation in cells upon stress and the drugs currently evaluated in clinical trials for p53 reactivation for improved therapy in MDS and AML. In normal cells, p53 protein is activated by numerous stress conditions like, oncogene activation, telomere shortening, replication stress, DNA damage, or elevated levels of reactive oxygen species (ROS). Wild-type p53 is released from MDM2 and MDM4 by phosphorylation by ataxia telangiectasia mutated (ATM) or ataxia telangiectasia and Rad3-related protein (ATR). (ATM-mediated phosphorylation of c-Abl mediates the release of p73 from the complex with MDM2, not shown). Consequent p53 acetylation and protein accumulation allow activation of p53 (and p73) transcriptional function. Activated ARF binds directly to MDM2 upon oncogenic stress and shifts the conformation so that p53 is released from the complex and becomes activated. ROS-activated c-Jun N-terminal kinase (JNK) was also shown to phosphorylate and activate p53 and p73. In normal cells, the negative feedback loop between p53-MDM2 is responsible for p53 protein turnover. In cancer cells amplified *MDM2* prevents p53 accumulation and activation. Mutant p53 protein accumulates in large quantities in cancer cells and escapes the regulation by the p53-MDM2 feedback loop. Drugs targeting wild-type and mutant p53 appraised in clinical studies for MDS and AML are marked in bold. Modified from [63, 70, 74–78]. →  →  → tandem of arrows indicates a multi-step process. Created with BioRender.com

Wild-type p53 is involved in multiple processes enabling tumor suppression and efficiently drives cell death in a transcription-dependent manner under stress conditions (Fig. 2). Cancer cells typically contain abundant mutant p53 protein resulting from disruption of the p53-MDM2 negative feedback loop (Fig. 2). In addition, mutant p53 is stabilized in cells by interacting with heat-shock proteins (HSP) which halt the degradation by MDM2 and other E3 ubiquitin ligases [62]. Thus, mutant p53 seems a plausible target for the development of targeted therapeutics (reviewed in [63]). Mutant p53 has three oncogenic properties that contribute to tumorigenesis. Loss-of-function, caused by the inability to bind the wild-type p53 target genes essential for tumor suppression; dominant negative effect (DNE) by forming hetero-tetramers with wild-type p53 [34], p73 or p63 [64] and the gain-of-function (GOF) propensity [65–68] which heavily depends on the context and include, cooperation with other oncogenes like HIF1a to withstand the hostile hypoxic environment, promoting cytokine secretion, angiogenesis and persistent cell cycling [69, 70], escaping cell death, immune evasion and enabling DNA damage repair and fueling nutrients [71]. The mechanisms and the significance of the GOF of mutant p53 propensities in tumor progression and proliferation have been debated. For example, the expression of mutant trp53 proteins (including two hot-spot equivalent to human R248 and R273) in HSPCs derived from *TRP53*^*−/−*^ mice did not accelerate tumorigenesis when compared to mice with reconstituted HSPCs from *TRP53*^−/−^ mice [72]. It has been concluded that mutant trp53 proteins do not hasten lymphoma development in *TRP53*^−/−^ and *TRP53*^+/−^. Next, a recent study showed that the genetic removal of twelve different p53 mutants previously reported to exhibit GOF activity did not impact proliferation, or response to chemotherapeutics, nor had the effect on growth or metastasis [73].

The documented prevalence of *TP53* mutant variants in MDS and AML urges the development of a therapeutic approach that aims at the reactivation of mutant p53 to reinstate the wild-type p53 tumor suppression function in malignant cells. The evidence from pre-clinical studies supports the feasibility of mutant p53 reactivation in cancer cells for improved cancer therapy. The approaches which advanced to clinical trials include; refolding mutant p53 to wild-type-like conformation with small molecules, stabilizing the DNA core domain with Zn^2+^ chelators, mutant p53 degradation and gene therapies [79]. At the same time, reactivation of wild-type p53 through inhibiting the p53-MDM2 interactions is equally feasible, and several compounds are now tested in clinical studies [80, 81].

### Mutant p53 conformation correctors

#### PRIMA-1MET/APR-246/Eprenetapopt

The first compound reported to act as a mutant p53 conformation corrector discovered in the protein-based screen, was CP-31398. It belongs to the Michael acceptors group of compounds [79, 82]. In 2002 Wiman and colleagues discovered a small molecule PRIMA-1 which killed tumor cells dependent on missense mutant p53 [83]. PRIMA-1 is a soft electrophile used as a scaffold to synthezise PRIMA-1^MET^ (eprenetapopt, APR-246), a methylated analog [84]. Both PRIMA-1 and APR-246 are converted to methylene quinuclidinone (MQ) [85]. MQ binds to cysteines in the p53 core domain via Michael addition and corrects the conformation of mutant p53 to wild-type-like as detected using conformation-specific antibodies (Fig. 2) [86–88]. PRIMA-1/PRIMA-1^MET^ reactivates wild-type p53 protein activity and induces cell death in multiple cancer cell lines with different contact and structural p53 mutants, including R110L, V157F, R175H, L194F, R213Q/Y234H, G245V, R248Q, R273C, R273H/P309S, R280K, and R282W and in in vivo tumor models [89–91]. In addition to mutant p53 per se, APR-246 also targets cancer cells’ redox balance [79, 91, 92]. Eprenetapopt was also recently shown to have mutant p53-independent functions and to kill tumor cells regardless of *TP53* mutant status [93]. The outcomes observed in in vitro studies may depend on various factors, such as the number of cells used, the concentration of the drug applied, the time course of the proliferation/viability assay, and the type of tumor being studied. The authors of the study discuss these factors, which are also applicable to many drugs, even those that have already been approved.

The first-in-human, phase Ib clinical trial, in hematological and prostate cancers (NCT00900614), allowed to estimate the maximal tolerated dose and clinical response was observed in several patients and one patient with *TP53*-mutated AML showed a reduction of blast percentage from 46% to 26% in the bone marrow [94] (Table 1).

**Table 1:** p53 reactivating drugs in clinical studies in MDS and AML (for complete list of clinical trials refer to the text)

p53 reactivating Mechanism drug		Clinical trial
Eprenetapopt (APR-246)	Mutant p53 refolding, restoring wild-type p53 transcription activity and cell death program	**Phase Ib NCT00900614**: Safety Study of APR-246 in Patients With Refractory Hematologic Cancer or Prostate Cancer^1^ **Phase Ib/II NCT03072043**: Safety and Efficacy of APR-246 w/Azacitidine for tx of *TP53* Mutant Myeloid Neoplasms^1^ **Phase Ib/II NCT03588078:** Study of the Safety and Efficacy of APR-246 in Combination With Azacitidine^1^ **Phase II NCT03931291**: APR-246 in Combination With Azacitidine for *TP53* Mutated AML (Acute Myeloid Leukemia) or MDS (Myelodysplastic Syndromes) Following Allogeneic Stem Cell Transplant^1^ **Phase III NCT03745716**: APR-246 & Azacitidine for the Treatment of *TP53* Mutant Myelodysplastic Syndromes (MDS)^1^ **Phase I NCT04214860**: APR-246 in Combination With Venetoclax and Azacitidine in *TP53*-Mutant Myeloid Malignancies^1^
Trisenox (ATO)	Mutant p53 refolding (structural mutants), restoring transcription wild-type p53 activity and cell death program	**Phase I NCT03855371**: Combination of Decitabine and ATO to Treat AML/MDS Expressing a Classified Type of Mutant p53
Atorvastatin	Mutant p53 degrader	**Phase I NCT03560882**: A pilot trial of atorvastatin in p53-mutant and p53 wild-type malignancies
RG7112(RO5045337)	MDM2 inhibitor	**Phase I NCT00623870**: A Study of RO5045337 [RG7112] in Patients With Hematologic Neoplasms^1#^
Idasanutlin (RG7388)	MDM2 inhibitor	**Phase I/Ib NCT01773408**: A Study of RO5503781 as a single agent or in combination with cytarabine in participants with acute myelogenous leukemia^#^ **Phase III NCT02545283**: Study of Idasanutlin With Cytarabine Versus Cytarabine Plus Placebo in Participants With Relapsed or Refractory Acute Myeloid Leukemia (AML) (MIRROS)^§^
Navtemadlin (AMG-232)	MDM2 inhibitor	**Phase I NCT02016729**: Study Evaluating AMG 232 Alone and in Combination With Trametinib in Acute Myeloid Leukemia^1^
Milademetan (DS-3032b)	MDM2 inhibitor	**Phase I NCT02319369**: Safety, Tolerability and pharmacokinetics of milademetan alone and with 5-azacitidine (AZA) in acute myelogenous leukemia (AML) or high-risk myelodysplastic syndrome (MDS)^1^ **Phase I/II NCT03634228**: Milademetan Tosylate and Low-dose Cytarabine with or without Venetoclax in treating participants with recurrent or refractory acute myeloid leukemia^@^
Siremadlin (HDM201)	MDM2 inhibitor	**Phase I NCT02143635**: Study to Determine and Evaluate a Safe and Tolerated Dose of HDM201 in Patients With Selected Advanced Tumors That Are *TP53*wt
APG-115	MDM2 inhibitor	**Phase Ib/II NCT04358393**: A Study of APG-115 Alone or Combined With Azacitidine in Patients With AML, CMML, or MDS
Sulanemadlin (ALRN-6924)	MDM2/MDM4 inhibitor	**Phase I/Ib NCT02909972**: Safety Study of ALRN-6924 in Patients With Acute Myeloid Leukemia or Advanced Myelodysplastic Syndrome

Based on clinicaltrials.gov, accessed on 10.09.2023

^1^Trial completed, has results

^#^Clinical trials are discontinued due to adverse events

^§^The study was stopped for futility based on efficacy results at the interim analysis; no unexpected safety findings were observed

^@^This study was terminated early due to a lack of adequate response and did not move to the Phase II portion of the study

Two, phase Ib/II clinical trials with APR-246 have been concluded so far. Both studies were designed to recruit patients with *TP53* gene mutations based on the primary mechanism of action of the drug. One study, for the safety and efficacy of APR-246 in combination with azacitidine and to assess complete remission (CR) of the patients with *TP53*-mutated myeloid neoplasm alone and in combination with azacitidine (AZA, vidaza) (NCT03588078) [95] (Table 1), and one to determine the safety and recommended dose of APR-246 in combination with azacitidine as well as to see if this combination of therapy improves overall survival (OS) (NCT03072043) [96]. In the NCT03588078 trial, fifty-two *TP53*-mutated patients (34 MDS, 18 AML) were recruited. 80% of the patients had complex karyotype and median baseline mutant *TP53* VAF was 20%. In MDS patients an overall response rate (ORR) was 62%, including 47% CR, with a median duration of response at 10.4 months. In AML patients the ORR was 33% including 17% CR. Of the patients who responded, 73% achieved mutant *TP53* VAF < 5% determined by negativity of next-generation sequencing (NGS). The median follow-up was 9.7 months, median OS was 12.1 months in MDS patients, and 13.9. The combination was well tolerated and showed potentially higher ORR and CR rates, and longer OS than reported with AZA alone [95]. In the NCT03072043 trial, fifty-five patients with at least one *TP53* mutation were treated. 89% of patients had a complex karyotype and/or multihit, e.g. > 1 *TP53* mutation or deletion 17p/-17. The mutant *TP53* median VAF in peripheral blood was 21%. 96% of patients had at least one mutation in the DNA binding domain. Azacitidine and eprenetapopt resulted in a 71% ORR and 44% CRR in the intention-to-treat population (50% for patients with MDS) with a median OS of 10.8 months, comparing favorably with single-agent azacytidine [96].

In the follow-up, a phase II study was performed (NCT03931291) to investigate the efficacy and safety of APR-246 in combination with azacitidine for *TP53*-mutated MDS or AML patients as post-hematopoietic stem-cell transplantation (HSCT) maintenance therapy [97]. Patients were screened pre-HSCT and from fifty-five patients screened post-HCT, thirty-three were enrolled and were treated with eprenetapopt in combination with azacitidine. In total, thirty patients had mutant *TP53* detectable in the pre-HSCT sample. Among ten patients who completed all 12 treatment cycles and did not relapse, pre-HSCT mutant *TP53* was detected in four patients and VAFs remained low during the treatment. At a median follow-up of 14.5 months, the median relapse-free survival (RFS) was 12.5 months. With a median follow-up of 17.0 months, the median OS was 20.6 months. It has been concluded that post-HSCT maintenance with eprenetapopt plus azacitidine was well tolerated with acceptable safety and may improve outcomes in mutant *TP53* MDS or AML [97].

Phase III clinical trial (NCT03745716) was conducted to compare the rate of CR and duration of CR, in patients with *TP53*-mutated MDS who will receive APR-246 and azacitidine or azacitidine alone. In total 154 patients were recruited. At a median follow-up of 12 months, Aprea Therapeutics, the study sponsor, reported that the CR rate was 37% higher in eprenetapopt with AZA arm compared to AZA alone but did not reach statistical significance and the study failed to meet the primary endpoint [98, 99].

Phase I trial (NCT04214860) was designed for dose-finding and cohort expansion study to determine the safety and preliminary efficacy of APR-246 in combination with venetoclax and azacitidine in patients with *TP53*-mutated myeloid malignancies. In total forty-nine patients were enrolled on the trial. Of the 49 patients who received study treatment, 20 (41%) had therapy-related acute myeloid leukaemia or had therapy-related secondary acute myeloid leukaemia, 24% of patients had more than one mutation of *TP53* and 80% of patients had VAF > 50%. The overall response rate among patients receiving eprenetapopt and venetoclax with azacitidine was 64%, and the CRR was 38%. In NGS-tested patients the clearance of *TP53* VAF < 5% was achieved in 26% of patients. The study showed that the combination of eprenetapopt and venetoclax with azacitidine had an acceptable safety profile in patients with previously untreated *TP53*-mutated acute myeloid leukemia [100].

### Arsenic trioxide/ATO/Trisenox

Arsenic trioxide (ATO) is a standard of care in acute promyelocytic leukemia. ATO refolds p53 structural mutants to wild-type-like conformation and induces p53 transcription activity and cell death. The crystal structure of ATO-bound mutant p53 proteins showed, that alike APR-246, ATO binds covalently cysteine residues in the DNA binding domain, yet specifically targets residues in the allosteric cryptic site composed of three cysteines C124, C135 and C141 [88]. Phase I clinical study (NCT03855371), a combination of decitabine and ATO to treat AML/MDS expressing a classified type of mutant p53, evaluates the side effect and treatment potential of DAC + ATO in *TP53* mutated high-risk MDS patients. According to the trial description, about two hundred AML/MDS patients will be recruited for *TP53* sequencing. The mutant p53-positive AML/MDS patients will be treated with the combination.

Phase II study (NCT03381781) decitabine, cytarabine (Ara-C) and arsenic trioxide (ATO) in the treatment of acute myeloid leukemia with p53 mutations is designed to sequence one thousand five hundred MDS/AML patients and randomize around one hundred patients with *TP53* mutations for the treatment. The outcomes of the studies remain to be reported.

### Mutant p53 degraders

The most clinically investigated mutant p53 degraders are HSP90 inhibitor ganetespib and the FDA-approved histone deacetylase inhibitor, vorinostat. Yet, neither of the drugs is studied in patients based on stratification dependent on the presence of mutant *TP53*. Only one trial reported outcomes for vorinostat and decitabine in patients with acute myeloid leukaemia or myelodysplastic syndrome, but did not report the status of *TP53* [101].

Atorvastatin, is a statin approved by the FDA to prevent cardiovascular disease in patients with abnormal levels of lipids. Statins block the key enzyme in the mevalonate pathway (sterol synthesis pathway) and lower cholesterol levels. Blocking the 3-hydroxy-3-methylglutaryl-CoA reductase (HMG-CR) prevents the synthesis of mevalonate, a cholesterol precursor, which when inhibited prevents protein prenylation, G proteins signaling and epithelial-to-mesenchymal transition (EMT) [102]. Atorvastatin, and other statins, were shown to promote degradation of misfolded mutant p53 proteins by releasing it from the complex with HSPs and the consequent degradation by E3 ligase CHIP (Fig. 2) [103]. Even though statins have demonstrated multiple mechanisms by which they might affect tumor cells’ viability, Phase I trial (NCT03560882), a pilot trial of atorvastatin in *TP53*-mutant and *TP53* wild-type malignancies, will determine if atorvastatin will decrease the levels of conformational mutant p53 in solid tumors and relapsed AML. The trial is currently ongoing.

Other p53 structure correctors which reached the clinical trials’ testing include COTI-2 [87] or PC14586, a small molecule p53 reactivator that is selective for the p53 Y220C mutation [104], yet these drugs are not being evaluated in MDS or AML patients and therefore will not be discussed in detail in this review.

APR-246/eprenetapopt has been tested or is tested in thirteen clinical trials (clinicaltrials.gov, accessed 10.09.2023) in cancer and is, so far, the most clinically advanced and promising drug reactivating mutant p53 in myeloid malignancies to date. The clinical trials showed that eprenetapopt has a favorable safety profile. It exhibits antitumor activity even against tumors that carry biallelic *TP53*-mutant clones. The clearance of p53 protein by immunohistochemistry correlates with clone size, which is measured by its VAF. This correlation can be used as a surrogate marker to determine the clinical activity of the tested compound [98].

The phase III clinical study with frontline APR-246 in combination with azacitidine in *TP53* mutant MDS showed good CR rates, yet the study did not meet the primary endpoint. The lack of difference between the arms could be attributed to the small effect size for APR-246 + AZA arm. This means that to see a statistically significant therapeutic effect, more patients may need to be treated. Additionally, patient selection based on factors beyond just the presence of a *TP53* mutation may be necessary. For instance, the type of *TP53* mutation could be a consideration, as discussed previously [98].

### Targeting p53/MDM2/MDM4 axis with small molecules

In tumors which retain wild-type *TP53*, p53 protein is inactivated through two major routes: through binding of MDM2/MDM4 oncoproteins to the N-terminal domain and inhibition of p53 transcription function, and MDM2-, ubiquitin-mediated proteasomal degradation (Fig. 2) [105].

In hematological malignancies, amplification of *MDM2* was reported for AML, CML ALL with no concomitant mutations in exons 4–10 of the *TP53* gene [106] and overexpression of MDM2 is associated with poor prognosis in AML [107]. MDM4 negatively regulates p53 and it was assessed in AML and MDS only in one study. Immunohistochemistry showed overexpression of MDM4 in 78 AML cases (92%) and 12 MDS cases (52%) and might be a therapeutic target in AML and MDS [108]. Targeting the interactions between p53-MDMD2 has become a feasible strategy after the identification of the key p53 residues fitting into the MDM2 hydrophobic pocket in the crystal structure analysis which showed three sub-pockets within the MDM2 hydrophobic cleft that are occupied by the Leu26, Trp23, and Phe19 amino acid side chains of p53 [109]. The first drug ever developed to target the p53-MDM2 protein complex was nutlin, a cis-imidazoline [110].

### RG7112 and Idasanutlin/RG7388/RO5503781

RG7112 (RO5045337), is a cis-imidazoline, a derivative of nutlin and the first MDM2 antagonist tested in clinical trials. It showed clinical activity in AML patients in the Phase I trial (NCT00623870) (Table 1). A total of 116 patients were enrolled and at least 16 patients with wild-type p53 AML were treated. *TP53* mutational analysis showed *TP53* mutations in 19 of 96 patients tested and most mutant *TP53* patients failed to show evidence of response. Ten genes, all p53 targets, were induced in wild-type p53 patients after treatment [111]. Yet, gastrointestinal toxicity, myelosuppression, and related complications resulted in the discontinuation of RG7112 clinical trials (reviewed in [112]).

Idasanutlin (RG7388, a selective MDM2 inhibitor) is widely studied in clinical trials. Idasanutlin is a pyrrolidine with enhanced potency, selectivity, and bioavailability compared to RG7112. It has been tested in Phase I/Ib trial (NCT01773408), a study of RO5503781 as a single agent or in combination with cytarabine in participants with acute myelogenous leukemia, yet, the final outcomes of the study were not published apart from the abstract [113]. Marker analysis of the patients enrolled in the study, using flow cytometry data for sixty-three evaluable patients showed that MDM2 expression in leukemic blasts was significantly associated with patients exhibiting a composite complete remission, and CR with incomplete hematologic recovery vs. no response. MDM2 per cent cell positivity in CD45^dim^/CD34 + /CD117 + leukemic blasts also showed an association with clinical outcomes. Overall, the analysis supports improved MDM2 antagonist clinical outcomes in AML patients with higher levels of MDM2 protein expression and thus, MDM2 protein expression from blasts may serve as a stratification biomarker for AML patients likely to benefit from idasanutlin-based therapy [114].

Phase Ib/II study (NCT03850535), evaluating the safety and efficacy of idasanutlin in combination with cytarabine and daunorubicin in patients newly diagnosed with acute myeloid leukemia (AML) and the safety and efficacy of idasanutlin in the maintenance of first AML complete remission, is designed, to evaluate the safety, efficacy, and the sponsor decided not to continue the study based on the overall company strategy in AML and a too-small group of patients enrolled.

In Phase III study (NCT02545283), study of idasanutlin with cytarabine versus cytarabine plus placebo in participants with relapsed or refractory acute myeloid leukemia (AML) (MIRROS), evaluated efficacy and safety of the treatment. A total of 447 patients were enrolled, 81% of patients had wild-type *TP53*. At the median duration of follow-up 6.7 months in both arms (drugs *vs* placebo), no subgroup showed a different outcome for OS. The median duration of CR was 13.9 months in the group with idasanutlin and 29.4 months in the placebo group. The myelosuppressive effect of idasanutlin was observed, yet prolonged neutropenia affected the response rates. The study did not meet the primary endpoint [115].

### Navtemadlin/AMG-232/KRT-232

AMG-232 is an improved derivative of piperidinone [116] and was evaluated in relapsed/refractory AML in a completed phase I study (NCT02016729) (Table 1) [117]. A study evaluated the safety and efficacy of AMG-232 alone and in combination with MEK inhibitor, trametinib. In the trial thirty-six patients with relapsed/refractory AML were enrolled, *TP53* mutational status was known for 44% patients at enrollment. Expression of *BAX*, *PUMA*, *P21*, and *MDM2* increased in the leukemic bone marrow and four patients achieved remission [117]. Two more studies are currently recruiting participants for the treatment of AML patients with navtemadlin; one phase Ib in combination with decitabine and venetoclax (NCT03041688) and one phase Ib testing the addition of an anti-cancer drug, navtemadlin, to the usual treatments (cytarabine and idarubicin) in patients with acute myeloid leukemia (NCT04190550).

### Milademetan/DS-3032b

Milademetan (DS-3032) tosylate hydrate is a specific and orally active MDM2 inhibitor [118]. Phase I study (NCT02319369) (Table 1) evaluated the safety, tolerability and pharmacokinetics of milademetan alone and with 5-azacitidine in acute myelogenous leukemia (AML) or high-risk myelodysplastic syndrome (MDS). Among 38 patients, two with AML and one with myelodysplastic syndrome had complete remission [112, 119].

In the phase I study (NCT03552029), patients were enrolled to milademetan plus quizartinib combination study in *FLT3-ITD* mutant acute myeloid leukemia (AML). Ten patients were only recruited, and the study was terminated based on a business decision by the Sponsor.

Phase I/II study (NCT03634228) (Table 1), milademetan tosylate and low dose cytarabine with or without venetoclax in treating participants with recurrent or refractory acute myeloid leukemia enrolled a total of 21 patients. Combination of MDM2 inhibition with inhibition of BCL2 potentiated apoptotic response however, no meaningful clinical responses were reported. Noticeable gastrointestinal toxicities were reported, and the study was terminated early due to futility [120].

### Siremadlin/ HDM201/CGM097

Siremadlin is a dihydroisoquinolinone derivative, the next-generation MDM2 inhibitor, evaluated in clinical studies [121]. Phase I (NCT02143635) (Table 1), a first-in-human dose-escalation study to determine and evaluate a safe and tolerated dose of HDM201 in patients with selected advanced tumors that are *TP53*wt, enrolled 115 patients with solid tumors and 93 patients with hematologic tumors (99% AML) [122]. A clear trend was observed for increases in serum GDF-15 (growth/differentiation factor-15), a biomarker for p53 transcription activity. Thirty-three per cent of evaluated patients had MDM2 amplification and fifty-three per cent of MDM2^amp^ patients achieved either partial response or stable disease. The drug showed an acceptable safety profile [122].

Two trials with siremadlin are currently recruiting participants with AML; one, phase I/II (NCT05447663) to evaluate siremadlin alone and in combination with donor lymphocyte infusion in acute myeloid leukemia post-allogeneic stem cell transplant, second, phase I/II (NCT05155709) a study of siremadlin in combination with venetoclax plus azacitidine in adult participants with acute myeloid leukemia (AML) who are ineligible for chemotherapy. The results remain to be published.

### APG-115

APG-115 belongs to spirooxindoles, a class of potent MDM2 inhibitors of K_i_ < 1 nM [123]. Phase Ib study (NCT04275518), of APG-115 single agent or in combination with azacitidine or cytarabine in patients with AML and MDS is recruiting one hundred two patients with relapsed/refractory AML and relapsed/progressed high/very high-risk MDS. Phase Ib/II study (NCT04358393)﻿ of APG-115 alone or in combination with azacitidine in patients with relapsed/refractory AML, CMML or MDS will enrol sixty-nine patients (Table 1). The outcomes of the studies remain to be published.

### Sulanemadlin/ALRN-6924

Sulanemadlin (ALRN-6924), is the first cell-permeating, stabilized α-helical peptide which mimics the N-terminal domain of the p53 and binds with high affinity to both MDM2 and MDM4 to activate p53 signaling in cancer cells [124].

Phase I/Ib (NCT02909972) (Table 1) safety study of ALN-6924 in patients with acute myeloid leukemia or advanced myelodysplastic syndrome has recruited fifty-five patients and evaluates anti-tumor effects of ALRN-6924 alone or in combination with cytarabine. The outcome of the study remains to be published.

The phase 1b breast cancer chemoprotection trial with ALRN-6924 in patients with p53-mutated breast cancer (NCT05622058) was terminated due to severe grade 4 neutropenia and alopecia, failing to meet the trial's main endpoints. Aileron Therapeutics, the drug owner, is running several other trials with ALRN-6924 and just recently offered the strategy to strengthen the NCT05622058 trial. The new strategy foresees using a lower dose of ALRN-6924 for chemoprotection than originally planned.

Currently, other MDM2 inhibitors are under investigation in clinical trials, like BI-907828 (brigimadlin) [125] but are not evaluated in MDS or AML. Yet, the list of clinical trials discussed above may urge us to conclude that targeting MDM2 with high-affinity inhibitors has not so far delivered the expected clinical benefit in patients with AML and MDS. Likely, other strategies are needed to overcome the persevering problem of the insufficient response of patients due to persistent neutropenia and adverse events related to the gastrointestinal tract.

One way to improve the patients’ outcomes with high-affinity MDM2 inhibitors might be their conjugation with antibodies to improve drugs’ selectivity or a design of less toxic combination strategy based on repurposed drugs like metformin. This well-known antidiabetic drug is currently being tested in a pilot, MILI clinical study in nondiabetic Li-Fraumeni Syndrome (LFS) patients with germline *TP53* variants (NCT01981525) [126]. The trial aims to assess the tolerability of daily oral metformin in LFS patients with the endpoint of reducing cancer incidence. The mechanism by which metformin might prevent or slow down cancer development in LFS patients is unclear. The mechanism may rely on inhibiting the pre-cancerous niche through a metabolic switch by activating AMPK, inhibiting mTOR, and improving insulin sensitivity in liver tissues of *TP53* mutant carriers [127]. Results from murine models of LFS showed significantly prolonged median overall survival after metformin administration, providing grounds for clinical trial initiation [128].

Drug repurposing emerges as a promising therapeutic approach in oncology since the drugs of potential anti-cancer activities have already been approved by the FDA for another indication and the safety profiles are known [129]. Multiple clinical studies, like the biomarker prevention trial studying the combination of metformin with aspirin in stage I-III colorectal cancer patients (ASAMET, NCT03047837) are ongoing. The outcomes of these trials have not been reported yet.

### Beyond MDM2/MDM4 inhibitors

Another promising strategy to target MDM2 is to promote its degradation using protein degrader, PROTAC. A recent pre-clinical study in breast cancer demonstrated the feasibility of reconstituting the p53 tumor suppressor pathway in the presence of mutant p53, through activation of p73 [130]. p73 belongs to the p53 protein family and together with p63 are ancestors of p53 in multicellular organisms. *TP73* gene is rarely mutated in cancers. Due to high structure and function homology, p73 protein recognizes a plethora of p53 target genes involved in tumor suppression but has also p53-independent functions. Stress-induced p73 post-transcriptional regulation appears to be similar to that of p53 [74, 131] and both proteins undergo regulation by MDM2/MDM4 (Fig. 2). In the case of p73 protein, ITCH E3 ligase and MDM2 are responsible for protein turnover [132]. p73 is emerging as a promising therapeutic target for improved cancer therapy in cancers with *TP53* gene mutations [129, 133]. Pharmacological reactivation of p73 in mouse models showed promising results with no apparent toxicity [130, 134]. We have shown that both, p53 and p73 proteins are reactivated by a repurposed drug, protoporphyrin IX, through targeting p53/MDMD2/MDM4 and p73/MDM2 interactions [135, 136]. While potently inducing cell death in tumor cells in pre-clinical studies, PpIX had only a mild effect on the proliferation of several normal cell lines/cells including human fibroblasts or peripheral blood mononuclear cells. Thus, PpIX or its analogs, might have better safety profiles in prospective clinical studies compared to MDM2 inhibitors. It remains to be investigated whether reactivating p73 for tumor suppression would yield comparable outcomes to wild-type p53 reactivation in terms of tumor suppression but with improved safety profile.

### The complexity of p53 protein biology in AML

Tuval et al., recently reported that the pre-leukemic clones with *DNMT3A* mutations have a selective advantage and an intrinsic chemoresistance as they pre-dominantly express pseudo-mutant p53[137]. The pseudo-mutant p53 protein is a misfolded wild-type p53 protein and has a limited transcriptional activity [138]. The protein exists in the equilibrium state in pre-leukemic blasts and was predominantly found in *DNMT3A*— mutated (wild-type *TP53*) AML enabling the clones’ enhanced self-renewal.

Refolding of pseudo-mutant with a structure-correcting peptide, pCAP-250, resulted in conformation refolding and restoration of p53 transcription activity in vitro and in vivo [137]. This implies that some sub-group of AML patients harbouring pseudo-mutant might profit from the therapy with p53 structure correctors rather than from the treatment with MDM2 inhibitors. Yet, due to the limited application of conformation-specific antibodies at the diagnosis, the stratification strategy allowing to distinguish between p53 wild-type-like conformation and unfolded conformation is not applied in the clinical study design.

The emerging importance of pseudo-mutant p53 in CH, requires modifications to the current model of *TP53*-mutated CH (Fig. 1b). Further studies are needed to evaluate the biology of pseudo-mutant in the pre-leukemic niche and to comprehend the co-existing factors contributing to clone evolution and fitness advantage in CH.

In conclusion, the role of p53 alterations in clonal hematopoiesis is still not fully depicted. The most advanced clinical drug, targeting p53 in hematological malignancies, is the mutant p53 reactivating compound, eprenetapopt (APR-246). So far, variable outcomes have been reported for MDM2 inhibitors and rational combination strategies may be crucial to enhanced efficacy with these compounds.

## Conclusions

There are other therapeutic strategies which are employed in the treatment of *TP53* mutated myeloid neoplasm and show promising outcomes. A recent multicenter observational study conducted on a large group of patients with the mutant *TP53* AML, has shown that the response to the standard induction therapy was only modest, and the overall survival rate was poor. The outcomes in this group of patients have not improved even with the application of novel therapies. However, the study revealed that those patients who responded well to induction therapy and proceeded to undergo allogeneic HCT had significantly better overall survival rates [139].

For eligible patients with mutant *TP53* AML, alloHCT in the first remission is recommended until better therapies are developed for improving long-term outcomes. Taking the current outcomes of the clinical trials of the p53-targeting therapies, it can be concluded that the management of the *TP53*-mutated myelodysplastic syndrome (MDS) and acute myeloid leukemia (AML) remains a therapeutic challenge despite significant recent advancements. Also, clinical studies on wild-type and mutant p53 reactivation have yielded inconsistent results in both MDS and AML.

Thus, further research is necessary to explore the biology of p53 in the pre-leukemic population of hematopoietic stem cells (HSCs).

The remaining open questions in this field are:

- Can the effectiveness of mutant p53 structure correctors in the *TP53*-mutated MDS and AML be determined through predictive biomarker stratification?
- Is there a possibility of high-affinity MDM2 inhibitors being approved as standalone treatments for wild-type *TP53* MDS and AML?
- Could the utilization of p53 reactivating compounds in combination therapies to impede key drivers potentially result in enhanced outcomes in MDS and AML?
- Is it possible to develop a biomarker discovery test that uses conformation-specific antibodies to stratify patients for mutant p53 reactivating drugs?

## Data Availability

No datasets were generated or analysed during the current study.
